# Efficacy and safety of isavuconazole for invasive fungal infections: A systematic review and meta-analysis of randomized controlled trials

**DOI:** 10.1093/mmy/myaf089

**Published:** 2025-09-27

**Authors:** Ayano Kawasaki, Ryuya Shintani, Risa Takao, Yuka Nakazawa, Takayuki Mihara, Shintaro Ikegami, Sora Shimada, Yuri Matsumoto, Yuko Okamoto, Yuki Igarashi, Yuki Enoki, Kazuaki Taguchi, Kazuaki Matsumoto

**Affiliations:** Division of Pharmacodynamics, Keio University Faculty of Pharmacy, 1-5-30 Shibakoen, Minato-ku, Tokyo 105-8512, Japan; Division of Pharmacodynamics, Keio University Faculty of Pharmacy, 1-5-30 Shibakoen, Minato-ku, Tokyo 105-8512, Japan; Division of Pharmacodynamics, Keio University Faculty of Pharmacy, 1-5-30 Shibakoen, Minato-ku, Tokyo 105-8512, Japan; Division of Pharmacodynamics, Keio University Faculty of Pharmacy, 1-5-30 Shibakoen, Minato-ku, Tokyo 105-8512, Japan; Division of Pharmacodynamics, Keio University Faculty of Pharmacy, 1-5-30 Shibakoen, Minato-ku, Tokyo 105-8512, Japan; Division of Pharmacodynamics, Keio University Faculty of Pharmacy, 1-5-30 Shibakoen, Minato-ku, Tokyo 105-8512, Japan; Division of Pharmacodynamics, Keio University Faculty of Pharmacy, 1-5-30 Shibakoen, Minato-ku, Tokyo 105-8512, Japan; Division of Pharmacodynamics, Keio University Faculty of Pharmacy, 1-5-30 Shibakoen, Minato-ku, Tokyo 105-8512, Japan; Division of Pharmacodynamics, Keio University Faculty of Pharmacy, 1-5-30 Shibakoen, Minato-ku, Tokyo 105-8512, Japan; Division of Pharmacodynamics, Keio University Faculty of Pharmacy, 1-5-30 Shibakoen, Minato-ku, Tokyo 105-8512, Japan; Division of Pharmacodynamics, Keio University Faculty of Pharmacy, 1-5-30 Shibakoen, Minato-ku, Tokyo 105-8512, Japan; Graduate School of Biomedical and Health Sciences, Hiroshima University, 1-2-3 Kasumi, Minami-ku, Hiroshima 734-8553, Japan; Division of Pharmacodynamics, Keio University Faculty of Pharmacy, 1-5-30 Shibakoen, Minato-ku, Tokyo 105-8512, Japan

**Keywords:** invasive fungal infections, isavuconazole, voriconazole, randomized controlled trial

## Abstract

**Background:**

Isavuconazole (ISA) is a treatment option for invasive fungal infections (IFIs) and is known for a favorable safety profile compared with other antifungal agents. However, comprehensive evidence regarding its efficacy and safety remains limited.

**Objectives:**

This study aimed to assess the efficacy and safety of ISA compared with other antifungal agents through a systematic review and meta-analysis restricted to randomized controlled trials (RCTs), to provide more reliable estimates of its clinical effects.

**Methods:**

Following Preferred Reporting Items for Systematic reviews and Meta-Analysis (PRISMA) guidelines, a comprehensive literature search was conducted using PubMed, the Cochrane Library, Web of Science, and ClinicalTrials.gov to identify RCTs comparing ISA with other antifungal agents. The primary outcomes were clinical response and mortality. Secondary outcomes included the incidence of adverse events, including serious, drug-related, and organ-specific toxicities. A subgroup analysis was conducted focusing on filamentous fungal infections, comparing ISA and voriconazole.

**Results:**

Three RCTs met the inclusion criteria. No statistically significant differences were observed between ISA and comparator agents in terms of clinical response, mortality, or total and organ-specific adverse events. A trend toward fewer adverse events was noted in the ISA group. In the subgroup analysis, ISA and voriconazole showed similar efficacy and overall safety; however, the incidence of both drug-related adverse events and hepatobiliary disorders was significantly lower in the ISA group.

**Conclusions:**

ISA demonstrated efficacy comparable to that of other antifungal agents, with a favorable safety profile in patients with IFIs, including filamentous fungal infections. This meta-analysis of RCTs provides high-quality evidence to support antifungal drug selection in clinical practice.

## Introduction

Invasive fungal infections (IFIs) are life-threatening, especially in immunocompromised patients.^1^ Conventional antifungals, such as voriconazole (VORI) and liposomal amphotericin B, are effective but are often limited by hepatotoxicity, nephrotoxicity, and drug–drug interactions.^2–4^

Isavuconazole (ISA), a third-generation triazole antifungal agent, offers several pharmacological advantages, including minimal interaction with cytochrome P450 3A4 (CYP3A4) ^5^ and the ability to be administered without dose adjustment in patients with renal impairment.^6^ ISA exhibits fungicidal activity against *Aspergillus* spp.^7^ and is considered a promising therapeutic option for filamentous fungal infections, including invasive aspergillosis and mucormycosis.^8^

According to current international guidelines, ISA is primarily positioned as an effective and safe alternative antifungal agent. The Infectious Diseases Society of America recommends ISA as an alternative to VORI for the treatment of invasive aspergillosis, particularly in patients at high risk of adverse effects or with concerns regarding hepatotoxicity.^9^ For mucormycosis, ISA is also recommended as an alternative when liposomal amphotericin B, the standard therapy, cannot be used. Similarly, the European Society of Clinical Microbiology and Infectious Diseases recommends ISA as an alternative to first-line therapy for invasive aspergillosis.^10^

IFIs progress rapidly and can become life-threatening within a short period; therefore, appropriate antifungal selection is critical to improving patient outcomes. However, previous meta-analyses have often included a mix of prospective and retrospective cohort studies, limiting the reliability of evidence regarding the efficacy and safety of antifungal agents.^11–13^ The present study aimed to evaluate the clinical response and adverse event rates associated with ISA through a systematic review and meta-analysis based exclusively on randomized controlled trials (RCTs), to provide more robust and reliable evidence for its clinical use.

## Method

### Study design

This study compared the efficacy and safety of ISA with those of other antifungal agents in patients with IFIs. The study design followed the PICO framework: the Population (P) included patients with IFIs; the Intervention (I) was treatment with ISA; the Comparison (C) was with other antifungal agents; and the Outcomes (O) assessed were efficacy and safety. The primary outcomes were overall clinical response and overall mortality. Secondary outcomes included the incidence of all treatment-emergent adverse events, serious adverse events, drug-related adverse events, and organ-specific events. This systematic review and meta-analysis were conducted in accordance with the Preferred Reporting Items for Systematic reviews and Meta-Analysis (PRISMA) guidelines.

A comprehensive literature search was conducted using PubMed, the Cochrane Library, Web of Science, and ClinicalTrials.gov. The search strategy combined free-text keywords related to IFIs, antifungal agents (including triazoles, amphotericin B formulations, and echinocandins), and the use of ISA. A detailed search query for PubMed is provided in Supplementary Table 1. The search included studies indexed in these databases up to September 17, 2024.

The exclusion criteria were studies in which ISA was used in combination with other antifungal agents, as well as preclinical studies and conference abstracts.

### Data extraction

Data extraction was performed independently by two reviewers. Any discrepancies were resolved through discussion and consensus agreement. The extracted data included study design (RCT), sample size, baseline characteristics of participants, details of the intervention and comparator groups, and outcome measures. To evaluate the comparative efficacy of ISA and VORI, a subgroup analysis was conducted using two RCTs that enrolled patients with filamentous fungal infections, such as *Aspergillus* and *Mucormycetes*. As the original publication did not clarify whether the reported number of hepatobiliary disorders referred to events or individual cases, Asahi Kasei Pharma Corporation was contacted and confirmed that the numbers represented individual patients.

To assess the methodological quality of each included study, the Risk of Bias tool version 1.0 was used, based on the Cochrane Handbook for Systematic Reviews of Interventions (Version 5.1.0). The following domains were evaluated: random sequence generation (selection bias), allocation concealment (selection bias), blinding of participants and personnel (performance bias), blinding of outcome assessment (detection bias), incomplete outcome data (attrition bias), selective reporting (reporting bias), and other potential sources of bias. Each domain was independently assessed by two reviewers and categorized as having low, high, or unclear risk of bias. The rationale for each judgment was based on information extracted from primary publications and their supplementary appendices. The certainty of evidence for each outcome was evaluated using the Grading of Recommendations, Assessment, Development and Evaluation (GRADE) methodology, taking into account factors such as risk of bias, inconsistency, indirectness, imprecision, and publication bias.

### Statistical analysis

Statistical analyses were conducted using R software (version 4.5.0; R Foundation for Statistical Computing, Vienna, Austria). Risk ratios (RRs) with corresponding 95% confidence intervals (CIs) were calculated. For the overall analysis, a random-effects model based on the DerSimonian–Laird method was employed due to clinical and methodological heterogeneity among the studies. In contrast, subgroup analyses limited to filamentous fungal infections comparing ISA and VORI were analyzed using a fixed-effect model based on the Mantel–Haenszel method, due to the relative homogeneity of the included trials. Statistical heterogeneity was assessed using the *I*² statistic.

### Ethics statement

No ethical approval was required as this is a review article with no original research data.

## Result

### Study selection

**Figure 1.**
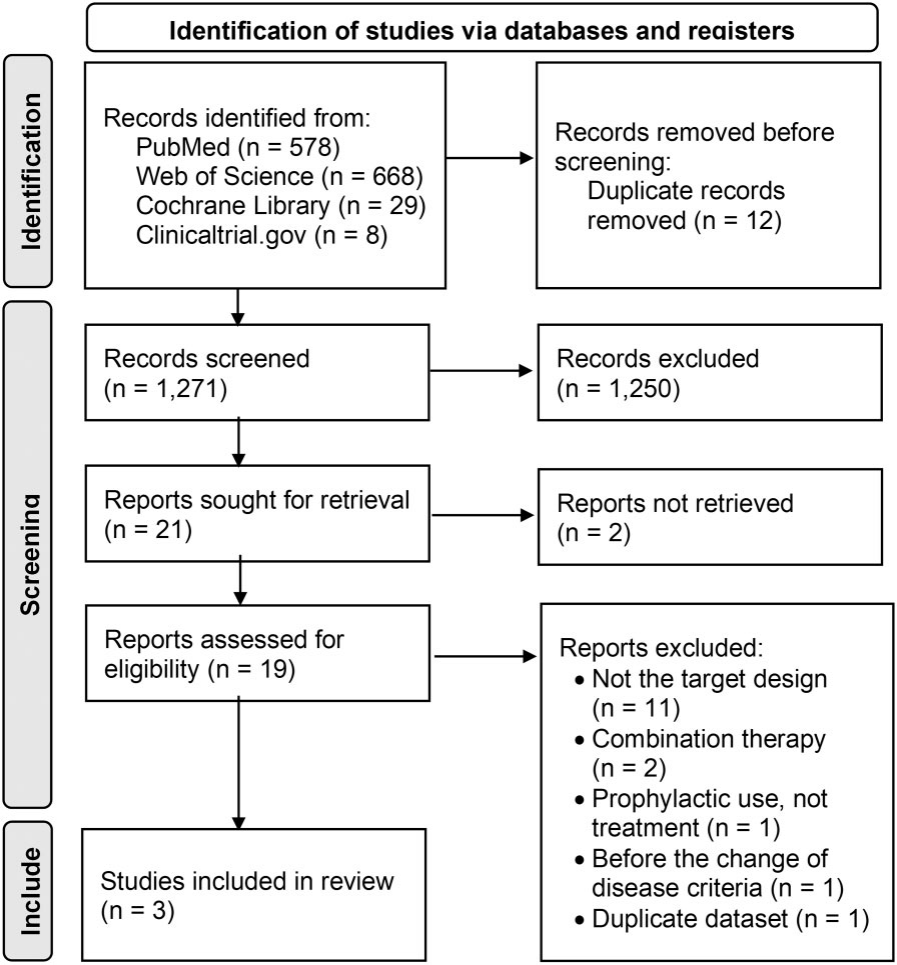
Flow diagram of study selection process for the meta-analysis.

A flowchart of the study selection process is presented in Fig. 1. A total of 1,283 records were initially identified through database searches. After removing duplicates, 1,271 unique records remained for screening. During the initial screening, 1,252 articles were excluded based on their titles and abstracts, as they did not meet the PICO criteria. In the second screening, 21 full-text articles were assessed for eligibility, and three studies were ultimately included in the analysis. Reasons for exclusion at the full-text screening stage included non-target study design (*n* = 10), use of combination therapy (*n* = 2), prophylactic use (*n* = 1), outcomes not meeting the inclusion criteria (*n* = 1), publication prior to updated disease definitions (*n* = 1), and duplicate data (*n* = 1).

### Study characteristics

Three RCTs were included in this analysis.^14–16^ The baseline characteristics of the study population are summarized in Table 1. In the study by Kullberg *et al*., 35% of patients in the ISA group and 40% in the caspofungin group were switched to oral antifungal therapy at some point after day 10.

**Table 1.** Characteristics of included RCTs.

Study	Design	Country, time interval	Sample size I/C	Fungus species	Dosing regimen	
					Intervention	Comparison
Maertens *et al*., 2018^15^	Post-hoc analysis of double-blind, global multicenter, comparative-group study	Global 2007–2013	164/140	*Aspergillus* spp. or other filamentous fungi	Isavuconazole 200 mg intravenously three times daily for 2 days, then 200 mg intravenously/orally once daily	Voriconazole 6 mg/kg intravenously twice daily on day 1, 4 mg/kg intravenously twice daily on day 2, then 4 mg/kg intravenously or 200 mg orally twice daily
Kullberg *et al*., 2019^16^	Randomized, double-blind, double-dummy, multicenter, noninferiority study	Global 2007–2015	220/220	*Candida* spp.	Isavuconazole *^a^* 200 mg intravenously three times daily for 2 days, then 200 mg intravenously once daily	Caspofungin *^b^* 70 mg intravenously once daily on day 1, then 50 mg intravenously (70 mg in patients > 80 kg) once daily
Kohno *et al*., 2023^14^	Randomized, multicenter, open-label study	Japan 2018–2021	55/28	*Aspergillus* spp. or *Mucorales*	Isavuconazole 200 mg intravenously three times daily for 2 days, then 200 mg intravenously or orally once daily	Voriconazole 6 mg/kg intravenously or 300 mg orally twice daily on day 1, then 4 mg/kg intravenously or 200 mg orally twice daily

a After day 10, at the investigator’s discretion, non-neutropenic patients could switch from intravenous to oral therapy.

b After day 10, at the investigator’s discretion, non-neutropenic patients could switch from intravenous caspofungin to oral voriconazole, beginning with a loading dose of 400 mg twice daily on the first day of oral therapy, followed by a standard dose of 200 mg twice daily thereafter.

### Outcomes

**Figure 2.**
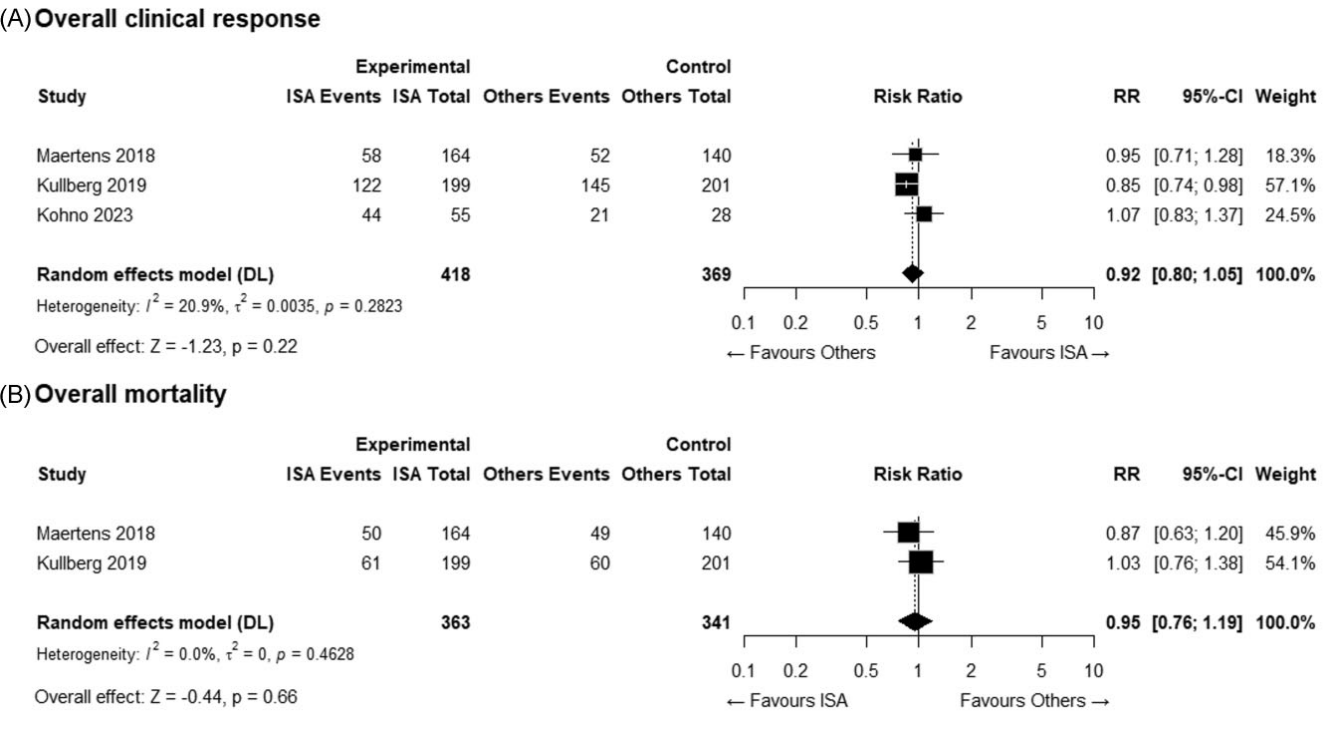
Forest plots of primary efficacy outcomes comparing isavuconazole (ISA) and comparator antifungal agents: (A) overall clinical response at the end of treatment and (B) overall mortality.

Regarding the primary outcomes, the overall clinical response rates were 53.6% and 59.1% in the ISA and comparator groups, respectively, with no statistically significant difference (Fig. 2A). Similarly, the overall mortality rates were 30.6% in the ISA group and 32.0% in the comparator group, also showing no significant difference (Fig. 2B).

**Figure 3.**
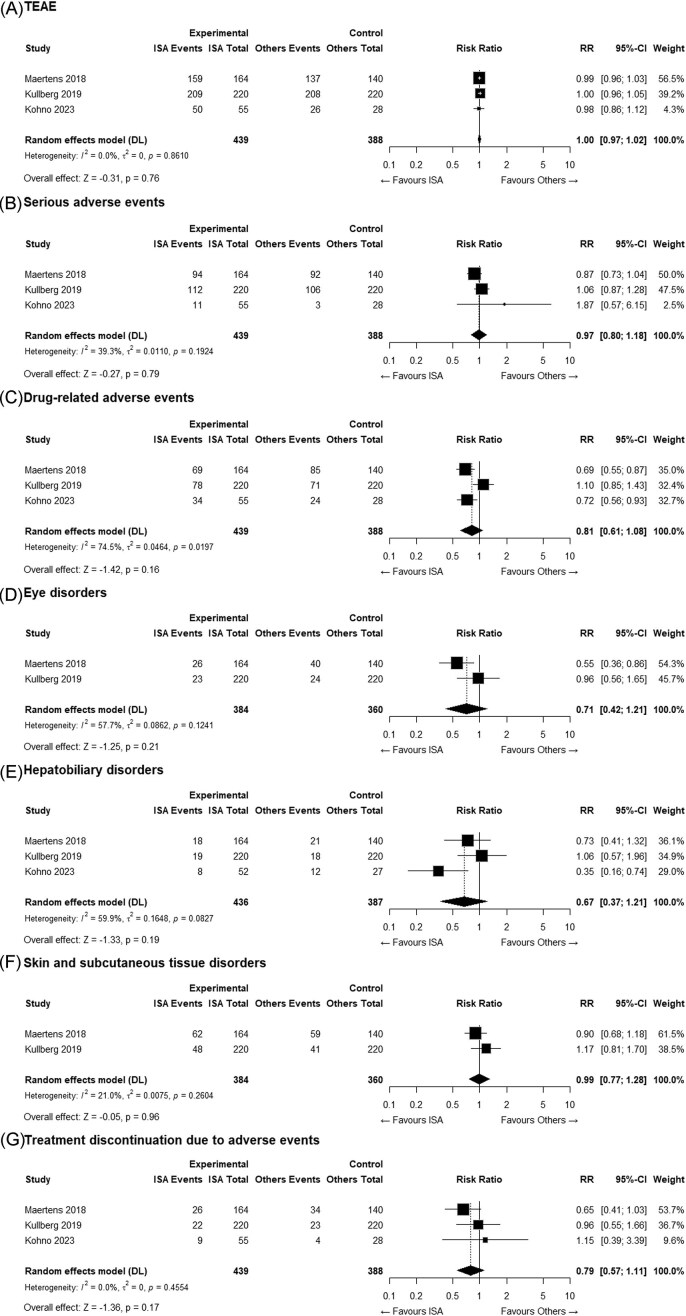
Forest plots of secondary safety outcomes comparing isavuconazole (ISA) and comparator antifungal agents: (A) all treatment-emergent adverse events; (B) serious adverse events; (C) drug-related adverse events; (D) eye disorders; (E) hepatobiliary disorders; (F) skin and subcutaneous tissue disorders; and (G) treatment discontinuation due to adverse events.

In terms of secondary safety outcomes, no statistically significant differences were observed between the ISA and comparator groups in the incidence of all treatment-emergent adverse events (95.2% vs. 95.6%, Fig. 3A), serious adverse events (49.4% vs. 51.8%, Fig. 3B), or skin and subcutaneous tissue disorders (28.6% vs. 27.8%, Fig. 3F). The pooled RRs for these outcomes were close to 1.0, indicating no substantial imbalance in the incidence of adverse events between the groups. Although the differences were not statistically significant, the ISA group consistently showed a lower incidence of drug-related adverse events (41.2% vs. 46.4%, Fig. 3C), eye disorders (12.8% vs. 17.8%, Fig. 3D), hepatobiliary disorders (9.6% vs. 10.8%, Fig. 3E), and treatment discontinuation due to adverse events (13.0% vs. 15.7%, Fig. 3G), suggesting a potential reduction in the risk of ISA-associated adverse events. None of the trials provided organ-specific reasons for discontinuation, preventing further analyses.

**Figure 4.**
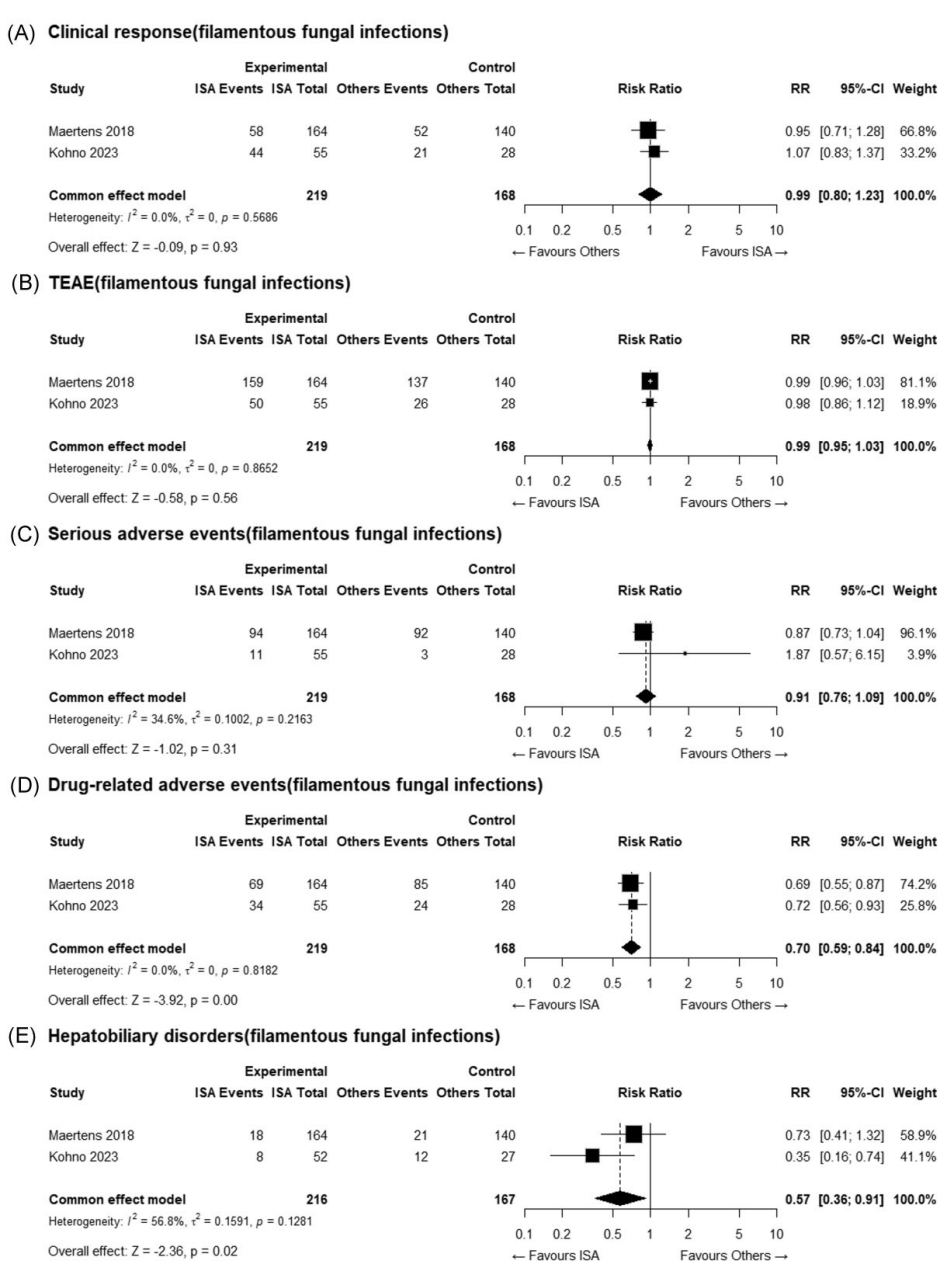
Forest plot of the subgroup analysis of patients with filamentous fungal infections comparing isavuconazole (ISA) and voriconazole (VORI): (A) overall clinical response; (B) all treatment-emergent adverse events; (C) serious adverse events; (D) drug-related adverse events; and (E) hepatobiliary disorders.

A subgroup analysis focusing on filamentous fungal infections, primarily caused by *Aspergillus* spp. and *Mucorales*, included two RCTs (Maertens *et al*. and Kohno *et al*.) that compared ISA with VORI. The overall clinical response rate was 46.6% in the ISA group and 43.5% in the VORI group, with no statistically significant difference, consistent with the findings of the overall analysis (Fig. 4A). Similarly, the incidence of all treatment-emergent adverse events was 95.4% in the ISA group and 97.0% in the VORI group, with no significant difference (Fig. 4B). The incidence of serious adverse events did not differ significantly between groups (47.9% vs. 56.5%, Fig. 4C). However, ISA demonstrated a statistically significant reduction in drug-related adverse events compared with VORI (RR: 0.70, 95% CI: [0.59; 0.84], Fig. 4D), indicating a broader safety benefit. Notably, for hepatobiliary disorders, ISA was associated with a significantly lower risk compared with VORI (RR: 0.57, 95% CI: [0.36; 0.91]), suggesting a potential safety advantage in this organ-specific outcome (Fig. 4E). Other outcomes could not be analyzed due to insufficient subgroup data.

### Risk of bias assessment

The risk of bias for the three RCTs included in this meta-analysis was assessed using the Cochrane Handbook for Systematic Reviews of Interventions criteria (Table 2). All studies were judged to have a low risk of bias for random sequence generation, allocation concealment, incomplete outcome data, and selective reporting.

**Table 2.** Risk of bias assessment for the included randomized controlled trials.

Entry	Maertens *et al*. 2018^15^	Kullberg *et al*. 2019^16^	Kohno *et al*. 2023^14^
Random sequence generation (selection bias)	Low risk	Low risk	Low risk
Allocation concealment (selection bias)	Low risk	Low risk	Low risk
Blinding of participants and personnel (performance bias)	Low risk	Low risk	High risk
Blinding of outcome assessment (detection bias)	Low risk	Low risk	Low risk
Incomplete outcome data addressed (attrition bias)	Low risk	Low risk	Low risk
Selective reporting (reporting bias)	Low risk	Low risk	Low risk
Other bias	Unclear risk	Unclear risk	Unclear risk

The studies by Maertens *et al*. and Kullberg *et al*. were conducted under double-blind conditions; therefore, they were assessed as having a low risk of performance and detection bias. In contrast, the study by Kohno *et al*. was open label; therefore, it was judged to have a high risk of performance bias. However, the outcome assessment in this study was conducted by an independent, blinded data review committee, resulting in a low risk of detection bias. All three trials were industry-sponsored (by Astellas, Basilea, and Asahi Kasei Pharma). As the extent of sponsor involvement in the study design and data interpretation was not fully disclosed, the “other bias” domain was rated as having an unclear risk.

The GRADE assessment demonstrated that the certainty of evidence was high for the primary outcomes and moderate for most secondary outcomes, primarily owing to heterogeneity or imprecision (Supplementary Table S2).

Given the limited number of included studies (*n* = 3), formal sensitivity analyses and assessments of reporting bias, such as funnel plots, were not conducted.

## Discussion

In this meta-analysis, no statistically significant differences were observed between ISA and other antifungal agents in the primary outcomes, overall clinical response rate and overall mortality, or in the secondary safety outcomes, including the incidence of overall and organ-specific adverse events. Although most safety outcomes did not reach statistical significance, adverse events generally tended to occur less frequently with ISA than with other antifungal agents. Notably, in the subgroup analysis focusing on filamentous fungal infections and comparing ISA with VORI, a significant reduction in the incidence of drug-related adverse events and hepatobiliary disorders was observed in the ISA group. These findings suggest that, although ISA demonstrates comparable efficacy to other antifungal agents, it offers a superior safety, particularly when used as an alternative to VORI.

Traditional treatment options such as VORI and liposomal amphotericin B are known for their strong antifungal efficacy; however, their clinical use is often limited by adverse effects, including hepatotoxicity, nephrotoxicity, and drug–drug interactions. These issues are particularly problematic in elderly patients, those requiring polypharmacy, and immunocompromised individuals, such as organ transplant recipients. ISA may offer important clinical advantages in such populations due to its relatively mild interaction with CYP3A4 enzymes and the absence of a need for dose adjustment in cases of renal impairment. Although visual disturbances are a known adverse effect of VORI, the present analysis did not reveal a significant difference in the incidence of ocular disorders. This may be explained by the inclusion of the study by Kullberg *et al*., which compared ISA with caspofungin rather than VORI, potentially attenuating the observed difference in this specific outcome.

Although previous pharmacokinetic studies have reported that ISA clearance is approximately 36% lower in Japanese individuals compared with Caucasians,^17,18^ no evidence of a significant impact of this pharmacokinetic difference on either efficacy or safety outcomes was observed in the RCTs included in this study, which involved Japanese participants.

Several meta-analyses have examined the efficacy and safety of ISA in treating IFIs; however, many included studies with heterogeneous designs, such as observational cohorts or prophylactic settings. In a network meta-analysis by Herbrecht *et al*.,^11^ four clinical trials were included, but only one involved ISA, highlighting the limited evidence available for this agent. The meta-analysis by Kato *et al*.^12^ incorporated studies conducted for prophylactic purposes, as well as retrospective cohort and case-control studies, raising concerns about heterogeneity in the observation periods and patient populations. Similarly, Weng *et al*.^13^ combined two RCTs with five retrospective cohort studies, resulting in substantial heterogeneity and an increased risk of bias due to limited control over confounding variables.

In all of these meta-analyses, the SECURE trial (Maertens *et al*., 2016) ^19^ contributed the largest patient population and carried the greatest statistical weight, potentially skewing overall conclusions toward its results. In contrast, the present study utilized a post hoc analysis^15^ of the SECURE trial that restricted the population to proven or probable cases based on the updated 2008 European Organisation for Research and Treatment of Cancer/Myco Spheres Group (EORTC/MSG) diagnostic criteria. This approach enhanced diagnostic specificity and excluded the confounding influence of possible cases. Moreover, the present meta-analysis included only prospective RCTs (*n* = 3), offering a more rigorous assessment and the highest level of evidence currently available for evaluating the clinical value of ISA.

Retrospective studies often lack adequate control of confounding variables and are more susceptible to bias, reducing the reliability of the evidence compared with RCT-based analyses.

In terms of efficacy, no statistically significant differences were observed between ISA and other antifungal agents with respect to the primary outcomes, including overall clinical response and mortality. These findings are consistent with those of prior studies and support the notion that ISA offers efficacy comparable to that of established antifungal agents.

Additionally, although the meta-analyses by Weng *et al*.,^13^ which included cohort studies, reported a statistically significant reduction in drug-related adverse events with ISA, the present overall analysis, which included trials comparing ISA with caspofungin, did not replicate this finding. However, in the subgroup analysis comparing ISA specifically with VORI in patients with filamentous fungal infections, a statistically significant reduction in drug-related adverse events was observed, highlighting a potential safety advantage of ISA over VORI in this context. By restricting the analysis to RCTs, the present study minimized the potential confounding and selection biases inherent in retrospective studies, thereby enabling a more accurate estimation of the treatment effect. These findings provide a more reliable evidence base to guide antifungal therapy selection in clinical practice.

Overall, the included RCTs had a low risk of major methodological bias, supporting the reliability of the findings. Although Kohno *et al*. conducted an open-label study, blinded outcome assessments mitigated these limitations. As all studies were industry-sponsored, a potential sponsorship bias should be considered.

This study has some limitations. First, all three included RCTs employed strict exclusion criteria, such as the omission of patients with severe organ dysfunction, profound immunosuppression, or contraindicated concomitant medications. These restrictions may limit the generalizability of the findings to broader real-world patient populations. Second, the number of eligible studies was limited, and the sample sizes were insufficient to conduct subgroup analyses for all outcomes, potentially reducing the statistical power of the results. Third, the included trials enrolled patients with different underlying diseases (invasive aspergillosis, chronic pulmonary aspergillosis, and invasive candidiasis), which may have introduced clinical heterogeneity and limited the generalisability of the pooled efficacy estimates. Fourth, our analysis relied on the 2008 EORTC/MSG definitions because all the included RCTs were designed and reported using these criteria. As the 2020 revised definitions were published after completion of these trials, it was not feasible to retrospectively apply them to the available data. Therefore, our findings should be interpreted in the context of the earlier definitions.

In conclusion, this meta-analysis, restricted to RCTs, demonstrates that ISA offers efficacy comparable to that of other antifungal agents, with a favorable safety profile in the treatment of IFIs, including filamentous fungal infections. Notably, in a subgroup analysis comparing ISA with VORI, ISA was associated with a significantly lower incidence of drug-related adverse events and hepatobiliary disorders. These findings provide high-quality evidence supporting the clinical use of ISA as a viable and potentially safer alternative, particularly in patients with contraindications to other first-line therapies or those at risk of hepatotoxicity.

## Supplementary Material

myaf089_Supplemental_File
