# Supplementary material for: Efficacy and safety of isavuconazole for invasive fungal infections: A systematic review and meta-analysis of randomized controlled trials
Source: Med Mycol. 2025 Sep 27;63(10):myaf089. doi: 10.1093/mmy/myaf089 (PMC12508740; doi:10.1093/mmy/myaf089)
Supplement: myaf089_Supplemental_File [file myaf089_supplemental_file.docx]

**Table S1. Search queries used for the systematic review** **across electronic databases.**

| ♯ | Search Query |
| --- | --- |
| 1 | deep mycosis |
| 2 | invasive fungal infection |
| 3 | mycoses |
| 4 | systemic fungal infection |
| 5 | disseminated fungal infection |
| 6 | fungemia |
| 7 | allergic bronchopulmonary mycosis |
| 8 | invasive mold infections |
| 9 | mucormycosis |
| 10 | cryptococcus |
| 11 | aspergillosis |
| 12 | candidiasis |
| 13 | voriconazole |
| 14 | posaconazole |
| 15 | fluconazole |
| 16 | ketoconazole |
| 17 | miconazole |
| 18 | itraconazole |
| 19 | ravuconazole |
| 20 | albaconazole |
| 21 | efinaconazole |
| 22 | fosravuconazole |
| 23 | fosfluconazole |
| 24 | oteseconazole |
| 25 | amphotericin B |
| 26 | liposomal amphotericin B |
| 27 | flucytosine |
| 28 | caspofungin |
| 29 | micafungin |
| 30 | anidulafungin |
| 31 | rezafungin |
| 32 | ibrexafungerp |
| 33 | nystatin |
| 34 | isavuconazole |
| 35 | isavuconazonium sulfate |
| 36 | cresemba |
| 37 | isavuconazonium |
| 38 | 1 OR 2 OR 3 OR 4 OR 5 OR 6 OR 7 OR 8 OR 9 OR 10 OR 11 OR 12 |
| 39 | 13 OR 14 OR 15 OR 16 OR 17 OR 18 OR 19 OR 20 OR 21 OR 22 OR 23 OR 24 OR 25 OR 26 OR 27 OR 28 OR 29 OR 30 OR 31 OR 32 OR 33 |
| 40 | 34 OR 35 OR 36 OR 37 |
| 41 | 38 AND 39 AND 40 |

**Table S2. GRADE summary of findings: certainty of evidence for each outcome in the meta-analysis**

| Outcome | Certainty of Evidence | Reasons of Downgrading |
| --- | --- | --- |
| Overall clinical response | High | No serious limitations |
| Overall mortality | High | No serious limitations |
| Treatment-emergent adverse events | Moderate | Small risk difference with high background event rate; downgraded for imprecision. |
| Serious adverse events | Moderate | Moderate heterogeneity |
| Drug-related adverse events | Moderate | High heterogeneity |
| Eye disorders | Moderate | Moderate heterogeneity |
| Hepatobiliary disorders | Moderate | Moderate heterogeneity |
| Skin and subcutaneous tissue disorders | High | No serious limitations |

GRADE, Grading of Recommendations, Assessment, Development and Evaluation
